# Self-Supervised Learning of Deep Embeddings for Classification and Identification of Dental Implants

**DOI:** 10.3390/jimaging12010039

**Published:** 2026-01-09

**Authors:** Amani Almalki, Abdulrahman Almalki, Longin Jan Latecki

**Affiliations:** 1 Department of Computer and Information Sciences, Temple University, Philadelphia, PA 19122, USA; latecki@temple.edu; 2 Department of Prosthetic Dental Sciences, Prince Sattam Bin Abdulaziz University, Al-Kharj 16278, Saudi Arabia; aa.almalki@psau.edu.sa

**Keywords:** classification, deep embeddings, transformer, masked autoencoders, dental implant

## Abstract

This study proposes an automated system using deep learning-based object detection to identify implant systems, leveraging recent progress in self-supervised learning, specifically masked image modeling (MIM). We advocate for self-pre-training, emphasizing that its advantages when acquiring suitable pre-training data is challenging. The proposed Masked Deep Embedding (MDE) pre-training method, extending the masked autoencoder (MAE) transformer, significantly enhances dental implant detection performance compared to baselines. Specifically, the proposed method achieves a best detection performance of AP = 96.1, outperforming supervised ViT and MAE baselines by up to +2.9 AP. In addition, we address the absence of a comprehensive dataset for implant design, enhancing an existing dataset under dental expert supervision. This augmentation includes annotations for implant design, such as coronal, middle, and apical parts, resulting in a unique Implant Design Dataset (IDD). The contributions encompass employing self-supervised learning for limited dental radiograph data, replacing MAE’s patch reconstruction with patch embeddings, achieving substantial performance improvement in implant detection, and expanding possibilities through the labeling of implant design. This study paves the way for AI-driven solutions in implant dentistry, providing valuable tools for dentists and patients facing implant-related challenges.

## 1. Introduction

In the 1980s, dental implants were introduced and have since become a global solution for patients with missing teeth [1]. Their impact on dental care has been significant, contributing to improved quality of life [2,3]. While implant treatments are now common, decades of clinical use have brought forth challenges, including complications in superstructures or implants and peri-implantitis [4,5]. Addressing these issues often requires additional prosthodontic, periodontic, or surgical interventions, necessitating detailed information about the intra-oral implant.

Accessing such information is straightforward when patients were previously treated at the same clinic, but complications arise when patients seek care elsewhere due to relocation or clinic closures. Dentists faced with limited data, such as oral photographs and radiographs, must identify crucial implant details, particularly the implant system, to proceed with treatments. While experienced dentists can navigate this process, those lacking sufficient knowledge face difficulties. Consequently, there is a demand for a system that can identify implant systems from limited data, irrespective of a dentist’s expertise.

Artificial intelligence (AI) technology, widely utilized in various fields, offers promising solutions. In medicine, AI has already proven valuable in robotics, medical diagnosis, statistics, and human biology [6]. Deep learning, a subset of AI, excels in tasks like prediction, object detection, and classification. Dentistry has seen the application of deep learning in diagnosing dental diseases from images, predicting treatments, classification, and statistical analysis [7,8,9,10,11,12,13,14]. Notably, deep learning-based object detection algorithms have enhanced diagnostic systems [15], often matching or surpassing human capabilities.

This study aims to develop an automated system using a deep learning-based object detection method to identify implant systems. The hypothesis is that this system can effectively detect and identify implants, offering a valuable tool for dentists and patients grappling with implant-related issues.

Automatic identification of dental implant systems has recently attracted increasing research attention, driven by the growing clinical demand to retrieve implant-specific information when historical treatment records are unavailable. Early approaches largely relied on manual comparison with manufacturer catalogs or expert knowledge, which is time-consuming and prone to error. More recent studies have explored deep learning-based solutions for implant detection and classification in panoramic and periapical radiographs, demonstrating promising results using convolutional neural networks and object detection frameworks [11,15,16]. However, these methods are typically trained in a fully supervised manner and require large amounts of labeled data, which is costly and difficult to obtain in dental imaging. Moreover, most existing approaches focus primarily on implant presence detection, with limited emphasis on implant system identification or design-level differentiation.

In parallel, self-supervised learning (SSL) has emerged as a powerful paradigm for medical image analysis, enabling models to learn meaningful representations from unlabeled data. Masked image modeling (MIM) methods, such as BEiT [17], MAE [18], and SimMIM [19], have shown strong performance in natural and medical imaging domains. Recent studies have successfully applied MAE-based self-supervised pre-training to medical image classification and segmentation tasks, including chest X-rays and dental imaging [20,21,22,23,24]. These approaches are particularly attractive in medical settings, where annotated data are limited and domain-specific characteristics differ significantly from natural images. Nevertheless, most existing MIM-based methods focus on pixel-level reconstruction, which may not be optimal for capturing high-level structural information critical for dental implant identification.

Motivated by these observations, we propose Masked Deep Embedding (MDE), a self-supervised pre-training framework that replaces pixel reconstruction with the prediction of deep patch embeddings. This design is better suited to learning discriminative representations for implant system identification and design analysis from dental radiographs, thereby addressing key limitations of existing supervised and self-supervised approaches.

Recent progress in self-supervised learning has highlighted the efficacy of masked image modeling (MIM) [17,19,20,21,25] as a pre-training strategy for Vision Transformer (ViT) [22,26] and the hierarchical Vision Transformer using shifted windows (Swin) [23,24,27,28,29,30]. MIM involves masking image patches and reconstructing them, allowing the network to deduce masked regions by utilizing contextual information. The capacity to aggregate contextual information is deemed crucial in the context of dental radiograph analysis. Among various MIM frameworks, the Masked Autoencoder (MAE) [25] stands out as a straightforward yet effective approach. MAE utilizes an encoder-decoder architecture, incorporating a ViT encoder that receives visible tokens and a lightweight decoder reconstructing masked patches using the encoder’s patchwise output and trainable mask tokens.

We advocate for self-pre-training, particularly advantageous when obtaining suitable pre-training data is challenging. Self-pre-training also eradicates domain discrepancies between pre-training and fine-tuning by unifying the training data [21]. Our experiments center on dental implant identification and classification in panoramic and periapical radiographs [16]. We apply our proposed method, Masked Deep Embedding (MDE) pre-training, on the same dataset used for the downstream task, i.e., the training dataset.

Specifically, we extend the self-supervised learning framework of the masked autoencoder (MAE) transformer [25]. While the MAE loss gauges the reconstructed masked patches’ quality, the MDE loss assesses predicted deep embeddings of masked patches. Post pre-training, the decoder is discarded, and the encoder is applied to the downstream task, i.e., dental implant detection. We compare three ViT Transformer initializations, including our proposed MDE, MAE [25], and a transformer without any self-pre-training. Experimental results demonstrate that MDE self-pre-training significantly enhances dental implant detection performance compared to the baselines.

Moreover, a comprehensive dataset addressing the simultaneous examination of implant design is currently unavailable. We contend that incorporating implant design amplifies the complexity of the computer vision problem, given the increased number of classes and potential class imbalances. Consequently, we enhance the existing dataset introduced in [16] under the supervision of a dental expert. We further enrich the dataset by creating annotations specifically for implant design, encompassing the classification of coronal, middle, and apical parts. The meticulous labeling process has resulted in a distinctive, high-quality augmented dataset. Interested parties can access our data, named Implant Design Dataset (IDD), upon request.

Our contributions are threefold:

- We replace MAE’s reconstruction of masked patches with the reconstruction of patch embeddings. Consequently, our loss is the simple $L_{1}$ distance between predicted and computed embeddings over masked patches.
- Our proposed method yields substantial performance improvement, surpassing all state-of-the-art methods in the dental implant detection task.
- The labeling of implant design extends the horizon of possible dental applications.

## 2. Dental Implant Design

**Figure 1:**
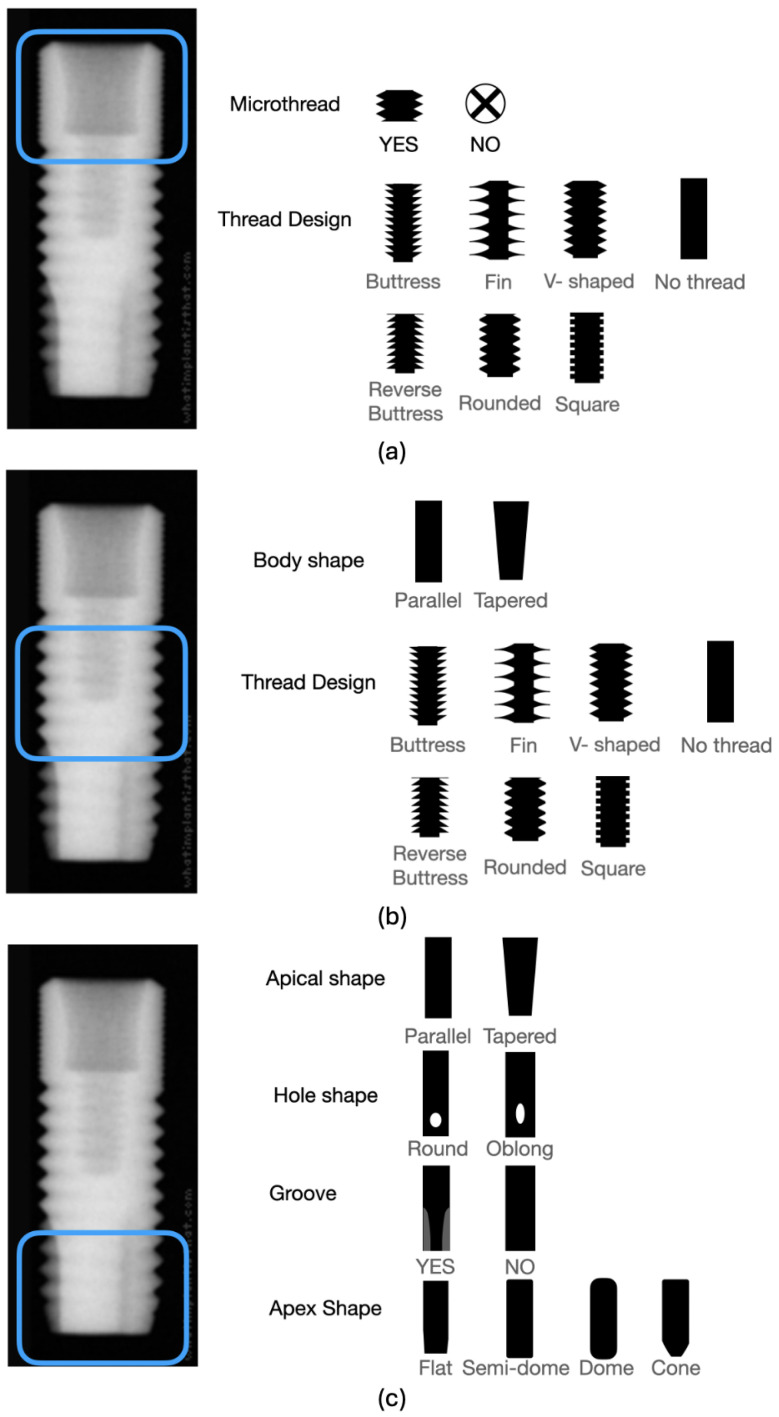
Categorization of (**a**) the coronal portion’s design based on bone or tissue level, the existence of microthreads, and the specific thread design, (**b**) the middle section’s design based on the shape of the body and the specific thread design, and (**c**) the design for the apical portion based on the shape of the apical hole, the configuration of the apical body, the presence of a groove, and the shape of the apex.

**Figure 2:**
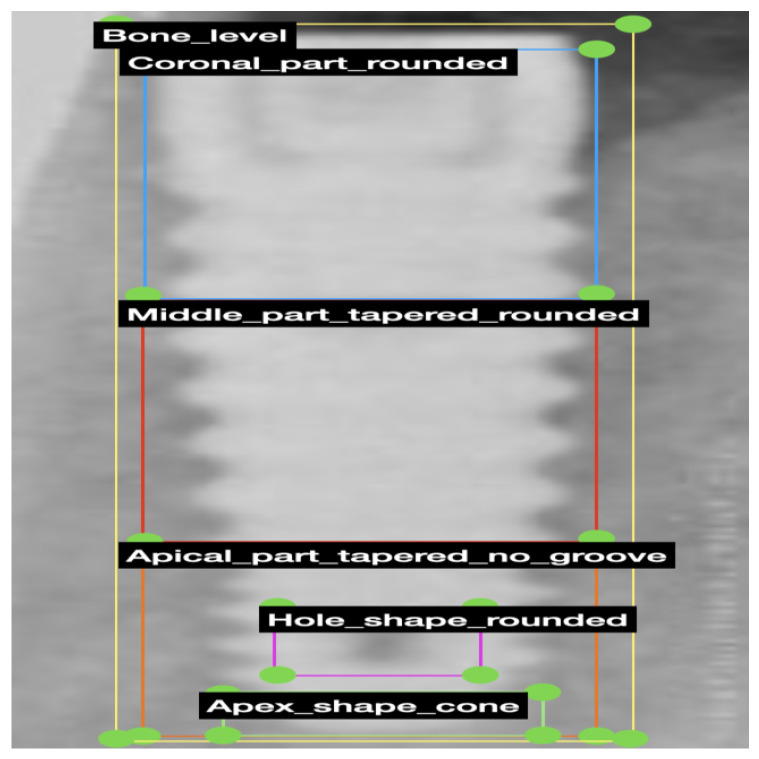
Image of a sample for categorizing and labeling implant design.

The categorization of implant design in the images was carried out, as detailed in Table 1. The coronal one-third of the implant underwent classification based on bone level, tissue level, microthread, and thread design (see Figure 1a). The middle one-third was categorized concerning body shape (straight or tapered) and thread design (see Figure 1b). The apical part was classified based on criteria such as the presence of a groove in the apical part, the shape of the apical hole, the shape of the apical body, and the apex shape (see Figure 1c). An experienced prosthodontist classified each group by referencing the manufacturer’s catalog and radiographs using the COCO-Annotator tool [31]. Subsequently, implant images were labeled according to the design classifications (see Figure 2).

## 3. Methods

### 3.1. Two-Stage Implant Detection Methodology

To address the task of dental implant detection, we propose a two-stage detection approach comprising the identification of individual implant design parts in the first stage and the subsequent inference of implant bounding boxes in the second stage. This method aims to enhance detection accuracy by breaking down the complex implant structure into distinct components before consolidating them into a comprehensive bounding box representation.

**Implant Design Parts Detection (First Stage).** In the initial stage of our methodology, we employ a dedicated object detection algorithm trained to recognize specific implant design parts. The chosen algorithm, in this case, is Mask R-CNN, which has been trained on an annotated dataset containing diverse dental implant images. The annotations include bounding box coordinates for each implant part such as the body, threads, and head.

During the inference process, the trained model scans input images, identifying the presence and localization of individual implant design parts. The output consists of bounding boxes for each detected component, providing a detailed spatial representation of the identified implant parts.

**Implant Bounding Box Inference (Second Stage).** Building upon the results of the first stage, the subsequent step involves inferring bounding boxes that encapsulate the entire dental implant structure. Post-processing techniques are applied to consolidate the detected implant design parts into a cohesive representation of the complete implant.

This involves:

- Handling Missing Implant Parts by developing post-processing strategies to infer or estimate missing parts based on the detected components. We implement techniques such as predictive models [32], spline interpolation [33], and adaptive thresholds [34] to enhance robustness in the presence of incomplete information.
- Analyzing spatial relationships between detected parts to refine the assembly process and improve the accuracy of the final representation.
- Employing clustering algorithms, such as K-Means Clustering [35], to group related implant design parts, adapting to variations in implant geometry, and aiding in the identification of missing components.
- Implementing heuristics based on known implant geometries to guide the assembly process, especially when dealing with missing parts.

For the clustering stage, K-means clustering is applied with the number of clusters set equal to the number of detected implant design parts within each image. K-means is initialized using the standard k-means++ strategy and is run for a maximum of 300 iterations. Post-processing thresholds for spatial grouping are empirically selected based on inter-part distance and overlap consistency and remain fixed across all experiments.

Upon successful grouping of individual implant parts, a bounding box is inferred to encapsulate the entire implant structure. This final bounding box serves as a holistic representation of the detected dental implant in the input image.

By dividing the detection process into these two stages and incorporating strategies to handle missing implant parts, our methodology aims to enhance the accuracy and robustness of dental implant detection, particularly in scenarios involving complex implant geometries and variations in image quality. The proposed approach provides a structured and systematic means of addressing the challenges associated with implant detection in diverse clinical contexts.

### 3.2. Self-Supervised Pre-Training with Masked Autoencoders

This section details the constituents of the Masked Autoencoder (MAE): the encoder, the decoder, and the associated loss function.

**Figure 3:**
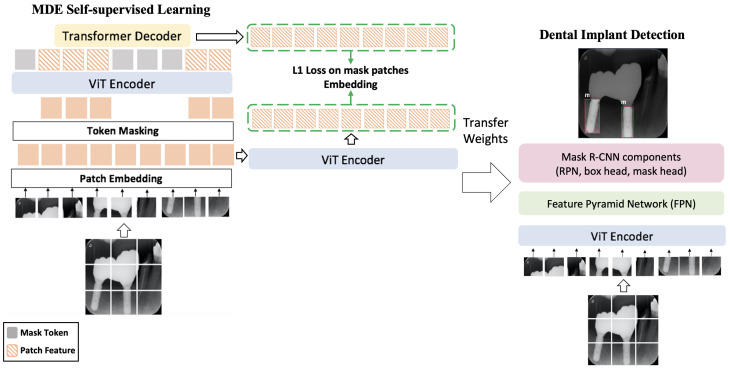
**Dental Implant Detection Pipeline with MDE Self-Pre-training.** The initial step in the dental implant detection pipeline for MDE self-pre-training involves dividing the input into non-overlapping patches. These patches undergo embedding using an MLP. Throughout the pre-training phase, the patch embeddings undergo random masking, and only the visible embeddings are employed by the transformer. Subsequently, the masked embeddings are merged with the encoded embeddings and directed to the decoder. The decoder’s role is to reconstruct the masked patches, followed by predicting the patch embeddings of these masked patches. The $L_{1}$ loss is employed to assess the similarity between the masked patch embeddings. Once pre-training is complete, the decoder is omitted, and the encoder is utilized as the backbone in Mask R-CNN with FPN for the detection.

**Encoder.** As illustrated in Figure 3(Left), the input undergoes partitioning into non-overlapping patches, randomly divided into visible and masked groups. The MAE encoder operates solely on visible patches, incorporating position embeddings to retain positional information. The resulting representation serves to reconstruct the masked input.

**Masked Sequence Generation.** Patch embeddings *E* are represented by a set. Following the MAE approach, a subset of patches is randomly masked, represented as $E_{m}$, and unmasked embeddings as $E_{um}$. Masked embeddings $E_{m}$ are replaced with a shared learnable mask embedding $E_{mask}$. Corrupted embeddings $E_{c}$ are formed by combining $E_{um}$ with the sum of $E_{mask}$ and positional embeddings *p*, inputted into the encoder.

**Decoder.** The MAE decoder is fed with a complete set of tokens, including patch-wise representations from the encoder and learnable mask tokens. Integrating positional embeddings with input tokens, the decoder aims to restore each patch embedding within its masked position, serving as an auxiliary module exclusively for pre-training.

**Loss computation.** We propose computing the $L_{1}$ loss between original and predicted embeddings of masked patches, deviating from MAE’s mean squared error (MSE) in pixel space. As our experimental results demonstrate, this change leads to performance improvement. This is in accord with observations in [36] that predict deep embedding of patches instead of pixel values yields better generalization and performance improvements. Model optimization is performed exclusively in the embedding space using an $L_{1}$ loss; any reconstructed images shown in the manuscript are provided solely for qualitative illustration and are not used for supervision.

### 3.3. Architectures for Downstream Tasks

After completing self-pre-training with MAE, we attach a task-specific head for the subsequent task, namely, the detection of dental implants.

The pre-trained ViT weights are utilized to initialize the encoder for detection. The features from the ViT backbone are conveyed to both the neck (FPN [37]) and the detection head (Mask R-CNN) to facilitate bounding box regression and classification. We opt for the Mask R-CNN [18] framework, given its widespread use in object detection research. Subsequently, the entire network undergoes fine-tuning to execute the detection task.

## 4. Experiments

### 4.1. Dataset

Implants Image Dataset [16] is a dental panoramic and periapical X-rays dataset consisting of 5572 annotated images with ground truth detection labels of dental implants. Each image size is 416 × 416 pixels.

The dataset includes dental implants from multiple commercial systems, specifically Bego, Bicon, and ITI, which are among the most commonly used implant manufacturers in clinical practice. All annotations and evaluations reported in this study are limited to these implant systems.

We contribute to further expanding the dataset by developing bounding boxes for dental implant design parts, including the thread design, body shape, apical shape, hole shape, and apex shape. This process was done by a prosthodontist using the COCO-Annotator tool [31].

We believe this is the most inclusive dataset for dental implant identification and classification in dental radiographs. We are providing our data, upon request, under the name Implant Design Dataset (IDD).

Table 2 summarizes the class distributions for coronal, middle, and apical design categories, highlighting imbalance across several subclasses. To mitigate this, we applied class-weighted loss functions and experimented with focal loss to down-weight dominant classes. These strategies were selected to align with the expanded class taxonomy introduced in Section 2 and the increased granularity of our label set.

### 4.2. Evaluation Metric

In all our experiments, we divided the data into five sets, each comprising around 20% of the images. Among these, one set remains constant as the test dataset, containing 1116 images, while the remaining four sets, each with 1114 images, form the training and validation datasets using cross-validation. This procedure is iterated five times. We use the Average Precision metric to evaluate object detection models. All data splits are performed at the patient level, ensuring that images from the same patient do not appear across training, validation, or test sets, thereby preventing patient-level data leakage.

### 4.3. Implementation Details

We conducted our experiments using the PyTorch (version 2.0) framework [38] and trained them on Nvidia Tesla V100 GPUs. Throughout all experiments, the batch size remains consistent at 4456, which corresponds to the effective batch size used per epoch (i.e., the total number of training samples), without gradient accumulation. A linear warm-up phase is applied for 10 epochs, followed by cosine learning rate decay for the remainder of training. This learning rate scheduling terminology is used consistently throughout the manuscript. The AdamW optimizer [39] is employed in all instances.

**Data augmentation.** We apply noise addition up to 6% of pixels, horizontal and vertical flipping, and 90° rotation clockwise and counter-clockwise.

**MDE pre-training.** The base learning rate is established at 1.5 × $10^{-4}$, weight decay is set to 0.05, β1 is 0.9, and β2 is 0.95. A cosine decay learning rate scheduler with a warm-up period of 10 epochs is applied. We utilize a random Masked Image Modeling approach with a patch size of 16 × 16 and a mask ratio of 25%. Additionally, we employ a linear prediction head, targeting an image size of 416 × 416.

**Task fine-tuning.** For downstream tasks, we employ single-scale training. The starting learning rate is 0.0001, and the weight decay is set at 0.05.

All models are trained for a fixed number of epochs without early stopping. Pre-training epochs and mask ratios are explicitly reported in Section 5.4. Model selection is performed based on validation performance, and the same training protocol is applied consistently across all experiments.

## 5. Results and Analysis

### 5.1. MDE Reconstruction

**Figure 4:**
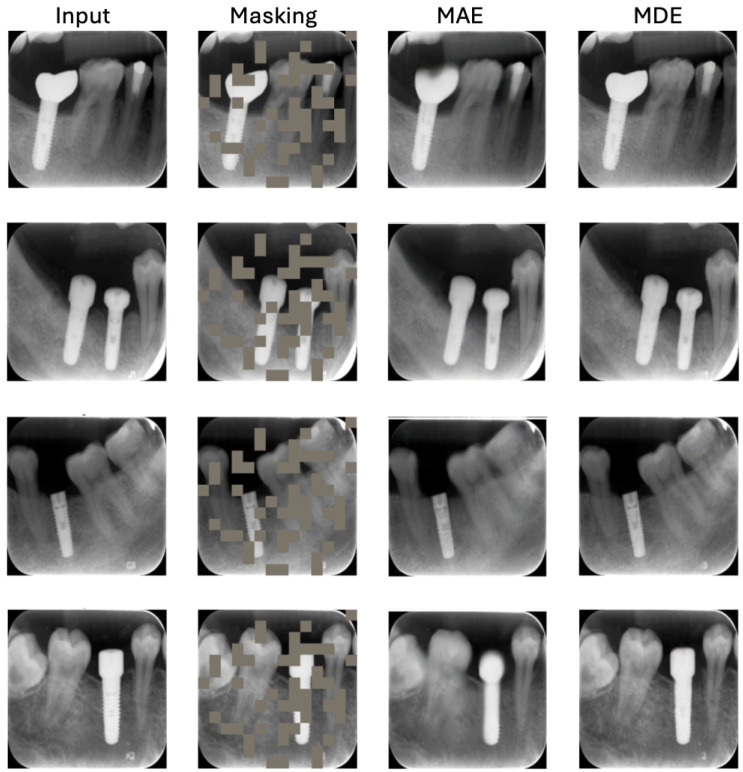
Results of MDE reconstruction. The first column displays the original images, while the second column shows the masked images, with gray patches indicating the masked regions. The third and fourth columns exhibit the reconstructions achieved through MAE and our MDE, respectively, from the unmasked patches.

The reconstruction outcomes of MDE are depicted in Figure 4. The figure comprises four columns illustrating the original images, masked images, images reconstructed using MAE, and images reconstructed using MDE. The results indicate that our approach excels in recovering missing information from the random context. It is important to emphasize that the primary objective of MIM is to enhance downstream tasks rather than produce reconstructions of the highest quality. It is worth noting that the process involves a reconstruction step. Instead of directly restoring pixel values from patches, the deep embeddings guide a generative model or decoding process to produce image samples. This generative model uses the high-level information encoded in the embeddings to create new pixel values, providing a reconstructed representation of the original images.

### 5.2. Dental Implant Classification and Identification

The presented tables provide a comprehensive overview of the results obtained in dental implant classification and identification tasks before and after the implementation of dental implant design labeling. The evaluation metric utilized is the ${AP}^{box}$ (average precision for bounding box detection), and various initialization strategies and backbones are compared.

In Table 3 (before the application of dental implant design labeling), the YOLOv5 model with CSPDarknet53 backbone achieved an ${AP}^{box}$ of 91.5, serving as a baseline for comparison. Notably, the ViT-B model with Random initialization, despite having no pre-training data, demonstrated a competitive performance with an ${AP}^{box}$ of 91.9, showcasing the ViT’s ability to learn meaningful features even in the absence of specific pre-training. The Supervised ViT-B model, pre-trained on ImageNet-1K with labels, improved the performance further to 92.6. The introduction of novel pre-training approaches, MAE and our MDE, both based on ViT-B and pre-trained on ImageNet-1K without labels, yielded impressive results of 93.2 and 94.9, respectively, with our MDE standing out as the most effective approach.

Moving to Table 4, where dental implant design labeling is employed, we observe consistent improvements across all models. The Random ViT-B model achieved an ${AP}^{box}$ of 92.4, showcasing the impact of incorporating dental implant design information. The Supervised ViT-B model experienced an increase to 93.2, emphasizing the value of labeled implant design data for pre-training. The MAE and our MDE models, both pre-trained on ImageNet-1K without labels, demonstrated substantial improvements, reaching ${AP}^{box}$ values of 94.0 and an impressive 96.1, respectively, with our MDE once again outperforming the other methods.

The results suggest that dental implant design labeling significantly enhances the performance of dental implant classification and identification models, regardless of the initialization strategy. Furthermore, the effectiveness of our MDE as a pre-training criterion is reinforced, indicating its robustness and suitability for the specific task at hand. These findings have important implications for the field of medical image analysis, underscoring the importance of domain-specific pre-training and the potential benefits of incorporating design information for more accurate and reliable dental implant detection. The combination of advanced pre-training techniques and domain-specific data augmentation can contribute to further advancements in the development of robust and precise models for dental implant recognition.

Table 5 reports the marginal contribution of each pipeline stage, including accuracy (AP) and computational cost (FPS). Stage-wise FPS is measured for (1) part detection and (2) final implant bounding-box inference, clarifying the source of overall performance gains.

### 5.3. Qualitative Results

**Figure 5:**
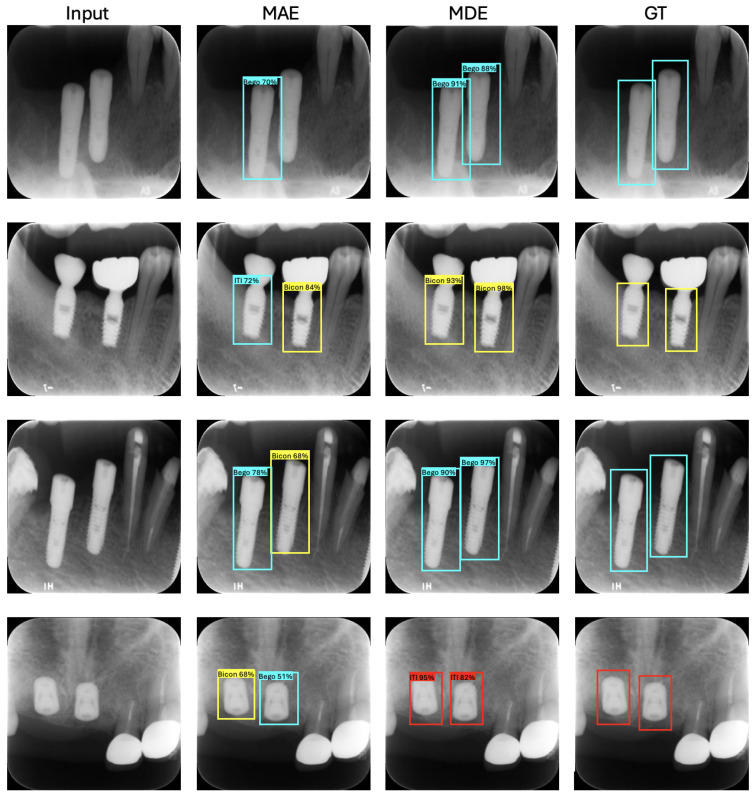
Qualitative results of dental implant detection and identification. The ViT pre-trained with the MAE approach exhibits missing or incorrect detections, whereas the ViT pre-trained with the MDE approach demonstrates accurate detection. Blue indicates the Bego dental implant system, yellow indicates the Bicon dental implant system, and red indicates the ITI dental implant system.

Figure 5 showcases qualitative samples highlighting the improved performance of dental implant detection and identification when using ViT pre-trained with MDE. The visual improvements align with the quantitative findings discussed in Section 5.2.

### 5.4. Parameter Setting

In Table 6, we conduct experiments focusing on dental implant detection with varying pre-training epochs and mask ratios for our MDE method. Firstly, we observe that extending the training duration does not lead to improved performance for MDE. Secondly, in contrast to the high mask ratio used in natural images [25], we find distinct preferences for mask ratios in downstream tasks related to dental implant detection. Notably, both tasks consistently exhibit enhancements as the mask ratio decreases from 65% to 25%. This improvement may be attributed to the fact that relevant features on dental X-rays tend to be smaller in size. The reported trends for mask ratio and training epochs were consistent across cross-validation folds; however, explicit uncertainty measures (e.g., mean ± SD or confidence intervals) are not reported and will be incorporated in future studies to further quantify the observed performance gains when reducing the masking ratio from 65% to 25%.

## 6. Conclusions

We have illustrated that the proposed MDE pre-training enhances state-of-the-art detection performance in the analysis of dental X-rays. Notably, MDE self-pre-training surpasses the performance of existing methods, particularly on a limited dataset, an aspect not previously explored. Our findings also indicate that parameters, such as mask ratio and pre-training epochs, should be customized when applying masked autoencoders pre-training to the domain of dental radiographs. These insights suggest that MDE has the potential to further enhance the already remarkable performance of ViTs in the analysis of dental X-rays. In our future work, we aim to assess the effectiveness of MDE pretraining in tasks related to prognosis and outcome prediction. We have also demonstrated that two-stage object detection with the first stage focused on domain-specific object parts like implant design parts can enhance object detection results. Although the proposed model is trained and evaluated on a fixed set of implant systems, the underlying self-supervised MDE framework is not inherently restricted to specific manufacturers. The learned representations capture structural and geometric characteristics of implants, suggesting that the approach can be extended to additional commercial systems given representative training data. Evaluating generalization to unseen implant brands and multi-center datasets remains an important direction for future work. An important limitation of the current study is the absence of external validation using data from independent institutions. Due to differences in imaging protocols, devices, and patient populations, such validation is essential to fully assess the robustness and clinical generalizability of the proposed method. While this was not feasible within the scope of the present work, evaluating the model on multi-center datasets will be a key focus of future research.

### Limitations

Despite the promising results, this study has several limitations that should be acknowledged. First, the annotation of implant design parts and system labels relies on expert supervision, which may introduce subjectivity and limit scalability. Second, the dataset includes a restricted number of implant manufacturers and images acquired from a limited set of sources, which may affect direct generalization to unseen commercial systems. Third, variations in image quality and acquisition conditions between panoramic and periapical radiographs may influence detection performance. Fourth, all experiments in this study are conducted using a single input resolution and a limited set of data augmentations to ensure controlled evaluation. Exploring multi-resolution training, patch-size sensitivity, and test-time ensembling to assess robustness and performance gains is left for future work. The robustness of the proposed method under diverse imaging conditions, including low-dose or low-contrast scans, motion and metal artifacts, and variability across imaging devices and fields of view, was not explicitly evaluated in this study and remains an important direction for future investigation. Fifth, the experimental comparison in this study focuses on YOLOv5 and ViT-based baselines to ensure fair architectural consistency and to isolate the impact of the proposed self-supervised pre-training strategy. While recent detection and transformer-based models could provide additional points of comparison, a broader benchmarking is left for future work. Sixth, although average precision (AP) is adopted as the primary evaluation metric, incorporating COCO-style AP size breakdowns, recall/mAR, class-specific metrics, and confusion matrices would provide a more detailed characterization of clinically relevant errors and is an important direction for future work. Seventh, although the quantitative results demonstrate strong performance, a detailed qualitative analysis of false positives and false negatives is not included. Future work will incorporate representative error visualizations and clinical interpretation of misclassification patterns to further improve model transparency and reliability. Eighth, although consistent trends were observed across folds, explicit uncertainty measures (e.g., standard deviations) are not reported and will be incorporated in future studies. Finally, the absence of external validation on multi-center datasets limits the assessment of robustness across different clinical environments. Addressing these limitations through larger, more diverse datasets and multi-institutional evaluation constitutes an important direction for future work.

## Figures and Tables

**Table 1:** Details of classes based on the classification of implant design.

Coronal	Middle	Apical
Bone level	Parallel fin	Hole round
Tissue level	Tapered fin	Hole oblong
Microthread	Parallel square	Parallel groove
Fin	Tapered square	Tapered groove
Square	Parallel no threads	Parallel no groove
No threads	Tapered no threads	Tapered no groove
V-shaped	Parallel V-shaped	Apex shape flat
Rounded	Tapered V-shaped	Apex shape cone
Buttress	Parallel rounded	Apex shape dome
Reverse buttress	Tapered rounded	Apex shape semi-dome
	Parallel buttress	
	Tapered buttress	
	Parallel reverse buttress	
	Tapered reverse buttress	

**Table 2:** Class distributions for coronal, middle, and apical implant design categories.

Design Category	Class	Count
Coronal	Bone level	1240
	Tissue level	870
	Microthread	410
	No threads	208
Middle	Parallel body	1935
	Tapered body	621
	V-shaped threads	382
Apical	Hole round	1710
	Hole oblong	545
	Apex cone	364
	Apex flat	188

**Table 3:** Results of dental implant classification and identification before employing dental implant design labeling.

Initialization	Backbone	Pre-Training Data	${\mathrm{AP}}^{\mathrm{box}}$
YOLOv5 [40]	CSPDarknet53	IN-1K w/Labels	91.5
Random	ViT-B	None	91.9
Supervised	ViT-B	IN-1K w/Labels	92.6
MAE	ViT-B	IN-1K	93.2
MDE (ours)	ViT-B	IN-1K	94.9

**Table 4:** Results of dental implant classification and identification after employing dental implant design labeling.

Initialization	Backbone	Pre-Training Data	${\mathrm{AP}}^{\mathrm{box}}$
Random	ViT-B	None	92.4
Supervised	ViT-B	IN-1K w/Labels	93.2
MAE	ViT-B	IN-1K	94.0
MDE (ours)	ViT-B	IN-1K	96.1

**Table 5:** Marginal contribution and computational cost of each pipeline stage.

Pipeline Stage	Description	AP Contribution (Δ)	FPS
Part Detection (Stage 1)	Detection of implant design parts	+2.1	32
Bounding-Box Inference (Stage 2)	Assembly of parts into final implant box	+1.3	58
Full Two-Stage Pipeline	End-to-end detection system	+3.4 (total)	28

**Table 6:** Impact of Mask Ratios on dental implant detection.

Mask Ratio	Pre-Training Epochs	${\mathrm{AP}}^{\mathrm{box}}$
65%	100	92.5
55%	100	93.2
55%	800	91.6
45%	100	94.0
35%	100	94.4
25%	100	94.9
15%	100	94.3

## Data Availability

The data presented in this study are available at https://universe.roboflow.com/al-xfpta/implants-meckw, reference number [16] (accessed on 20 February 2024).
